# Effects of Boric acid as Maternal Feed Additives on the Development and Sex Ratio of Mouse pups

**DOI:** 10.1007/s12011-024-04099-3

**Published:** 2024-02-12

**Authors:** M. B. Aykal, M. N. Gecin, I. Sogut, F. Kar, A. C. Taskin

**Affiliations:** 1 Department of Laboratory Animal Science, Aziz Sancar Institute of Experimental Medicine, Istanbul University, Istanbul, Türkiye; 2 Faculty of Medicine, Department of Biochemistry, Demiroglu Bilim University, Istanbul, Türkiye; 3 Department of Medical Biochemistry, Faculty of Medicine, Kutahya Health Sciences University, Kutahya, Türkiye

**Keywords:** Boric acid, Reproduction, Breeding, Sex ratio

## Abstract

Boron is primarily used in industrial applications, with recent interest revolving around its effects on metabolism. In this study, we administered boric acid (BA), which has positive effects on reproduction, in conjunction with feed supplementation to serve as a model for experimental animal development and breeding. The pregnancy performance, offspring development, and biochemical effects of mice given feed supplemented with BA at concentrations of 0 (control group), 250, and 500 ppm (BA groups) were investigated. A total of 18 female Balb-C mice were utilized for pregnancy. The mice were given the BA-supplemented feed during a period encompassing three weeks of pregnancy and three weeks of lactation. The numbers and weights of offspring born in cages on days 19–21 were determined. Blood and tissue samples were collected from the offspring during the third week postnatal, and the malondialdehyde (MDA) and total antioxidant and oxidant status (TAS, TOS, and OSI) levels were determined. A significant increase in female offspring was observed in the groups born to mice fed with BA compared to the control group. Positive development in organ weights was observed in the 250-ppm BA group. The 250-ppm group exhibited a significant increase in TAS compared to the control group, while TOS and MDA levels showed a decrease. Also, the levels of BA groups were found to decrease in both the OSI index serum and organ samples compared to the control group. Thus, the use of 250-ppm BA demonstrated positive effects on female offspring production, organ development, and antioxidant levels.

## Introduction

The chemical element boron (atomic number 5) typically exists in the environment in the oxidized state of boric acid (BA). Upon ingestion, it enters the bloodstream promptly and is excreted without accumulation. BA acts as a Lewis acid and plays a substantial role in the modulation of numerous enzymes. It also plays vital roles in functions such as cell growth, reproduction, development, and energy metabolism [1]. BA comprises approximately 17.5% boron [2]. In addition to being used as an antioxidant, anti-inflammatory, and anti-cancer agent, BA has been reported to have positive effects on embryonic development, bone development, the immune system, psychomotor functions, and cognitive functions [3]. Recent research has focused intensively on the potential beneficial effects of BA on rat Sertoli cells, mouse Leydig cells, and fetal embryo development [4].

In a study by Ince et al., rats fed with a gavage tube for 14 days with boron doses of 0.04 and 2.05 g after becoming pregnant showed improved gene expressions during the early embryonic period and enhanced fetal development [5]. Observations of fertilized trout eggs fed with boron in the range of 2.2 to 90.6 mmol/l indicated that boron supports development in a dose-dependent manner [6]. Adult Xenopus laevis fed with a diet containing 45, 310, and 1850 micrograms b/kg for 120 days showed abnormal development disorders in high-dose groups (310 and 1850 micrograms b/kg), while the low-dose group (45 micrograms) exhibited normal reproductive development [7].

Recent studies have been conducted on the antioxidant properties of BA under in vivo and in vitro conditions. Galleria mellonella larvae were subjected to various doses of BA as a supportive diet, resulting in increased superoxide dismutase (SOD) activity at doses of 156 and 620 ppm. However, at high doses, such as 1250 and 2500 ppm, antioxidant levels decreased, and larval and pupal mortality increased [8]. In a study investigating the protective effect of 2-ppm BA in human blood against aflatoxin B1 toxicity, it was found that BA reduced oxidative stress caused by aflatoxin by increasing the levels of antioxidants such as SOD, catalase (CAT), and glutathione peroxidase (GSH-PX) [9]. Furthermore, in another study determining the effects of BA injected into eggs at different doses on the bursa fabricius and spleen, low doses (1000-ppm BA) led to the involution of the bursa fabricius and, indirectly, to an increase in plasma cell count in the spleen [10]. Additionally, a study on the immune and antioxidant status and growth performance of lambs fed with diets containing and not containing sufficient Ca + showed that the addition of BA at 40 ppm increased total antioxidant activity and SOD1 gene expression [11].

Recently, there has been a focus on the potential beneficial effects of BA on rat Sertoli cells, mouse Leydig cells, and fetal embryo development [5].

While precision in ration preparations is generally applied to protein, metabolic energy, and macro elements, the necessary attention is not given to their levels of micro-elements. This is due to the complexity of the bioavailability of micro-elements and insufficient research on the subject [12]. In a study by Ince et al., rats fed with boron via a gavage tube for 14 days showed improved fetal development in the early embryonic period after becoming pregnant [5]. Our studies showed that BA influenced *vitro* mouse embryos and in vitro cryopreserved mouse embryos [13]. This study aims to apply BA to feed for its positive effects on reproduction using a feeding model for experimental animal development and rearing. The goal is to determine whether and to what extent feeding mice with BA-supplemented feed contributes to pregnancy performance and the development of born offspring.

## Materials and methods

### Animals

#### Ethical Approval

was obtained from the Animal Experiments Local Ethics Committee with decision number 2022/24 from Istanbul University. Experimental animals were obtained from Istanbul University Aziz Sancar Experimental Medicine Research Institute Laboratory Animals Department, and all care was provided in the laboratory belonging to the department. In this study, 18 female Balb-C mice with a live weight of 20–25 g were used as animal subjects. The mice were housed in standard cages with a 12-hour light and 12-hour dark rhythm, maintained at 22 ± 2 °C, and with relative humidity in the range of 45–65%. They were provided with European standard Type 1 cages from the Aziz Sancar Experimental Medicine Research Institute, and the cage equipment was replaced at each week for new bedding material and washed cages.

### Preparation of Feed with BA Addition

The amount of BA used in the experiment was determined as 0 (control group, 6 mice), 250, and 500 ppm (BA groups, 2 × 6 mice) (i.e., 0%, 0.025%, 0.05%; 18 mice). The feeds were prepared by the Arden Research & Experiment commercial feed company. The feed contained cereals, oilseeds, oils, starch-industry by-products, dicalcium phosphate, sodium carbonate, choline chloride, amino acids, vitamin and mineral premixes, antioxidant substances, mycotoxin binders, and probiotics. BA was not added to the control group. In the 250-ppm BA group, 250 ppm of BA per kg/diet was added and 500-ppm BA group, 500 ppm of BA per kg/diet was added raw materials were placed in a mixer, and after becoming homogeneous, the resulting mixtures were pelletized and placed in drying cabinets.

### Feeding Period

The (female) animals to be used in the experiment were mated with male mice of the same breed. During the mating (1 week), pregnancy period (3 weeks) and lactation period (3 weeks), they were fed with the pelleted feed described.

### Mating and Pregnancy Period

The body weight of each female mouse was recorded before mating. Grouping and intra-group numbering were performed. Pregnancy was detected on days 10–13 following mating, and the pregnant mice were individually placed in cages during the pregnancy period. The number of newborn pups and the date of birth were determined in cages where birth occurred between days 19–21.

### Postnatal Development Period

The number of surviving pups and the birth weights of the newborn pups were recorded on days 0–1 after birth. The number of surviving pups inside the cage was determined on days 7 and 14 after birth. On day 21, gender differentiation was performed, and the number of surviving pups was determined [14].

### Blood and Biochemical Evaluation

To assess the development of newborns, blood and tissue samples were taken from five female and five male mice from each of the three (one control and two BA) groups. Euthanasia was not performed on any pups. Blood was taken from the mice via intracardiac puncture under gas anesthesia (RWD), and serum was obtained by centrifugation. The obtained serum was stored at − 80 °C in the refrigerator. After blood collection, tissues were taken from euthanized pups, placed in liquid nitrogen for at least 1 h, and then transferred to a refrigerator, again at − 80 °C. Tissues were homogenized in ice-cold PBS (pH: 7.4) using a homogenizer. Homogenates were centrifuged at 600xg for 10 min (Rotina 380R Hettich, Tuttlingen, Germany). The malondialdehyde (MDA)(BT LAB Assay, Cat. No: E156Ra), total antioxidant (TAS) (Rel Assay Diagnostics Company Lot:EK21122A), and total oxidant (TOS) (Rel Assay Diagnostics Company Lot: 21135O) levels were measured in the supernatant. MDA levels (nmol/mg protein) were determined according to the method applied by Ohkawa [15]. TAS (mmol Trolox eq/L) and TOS (µmol H_2_O_2_ eq/L) levels were measured using a commercial kit (Rel Assay Diagnostics Gaziantep, Turkey), as previously described by Sogut et al. [3].

### Statistical Analyses

Statistical analyses were performed using the GraphPad Prism version 8.4. For data that showed compliance with the normal distribution, the analysis of variance (ANOVA) test using the post-hoc Tukey test was applied, while for data for which the normal distribution did not fit, the Kruskal–Wallis ANOVA with the post-hoc Dunn’s multiple comparison test was applied. Results were expressed as the mean ± SD and median (25% percentile – 75% percentile), *p* < 0.05was considered significant (Table 1).

**Table 1:** Chemical Analysis of Feed Composition

Moisture	Up to 12.5%
Crude Protein	At least 24.0%
Crude Fiber	Up to 5%
**Raw Materials: Soybean Meal**	**Minimum – Maximum 36–40%**
**Bonkalite**	**Minimum – Maximum 30–32%**
**Crude Ash**	**Up to 8.0%**
**Crude Fat**	**At least 5%**
**Ca**	**Minimum – Maximum 1.0–1.5%**
**P**	**Minimum – Maximum 0.7–0.8%**
**Na**	**At least 0.28%**
**Vitamin A (IU/kg)**	**At least 36,000**
**Vitamin D3 (IU/kg)**	**At least 6,500**

## Results

Following the mating and supply of feed, the postpartum offspring numbers were compared. In the control and the 250-ppm and 500-ppm BA groups, there were 55, 51, and 52 offspring, respectively (no significant difference; (*p* > 0.05) (Table 2). When weight measurements of the offspring were conducted on the 21st day, the control group had (9.5 gr.) female and (10) male individuals, while in the BA groups, these numbers were (11.5 gr) females and (10 gr males (250-ppm BA) and (7.5 gr females) and (8 gr males (500-ppm BA). No significant differences were observed among the groups in terms of weight gain (*p* > 0.05), although the 500-ppm group did display lower weight gain than the control group and 250 ppm BA (*p* < 0.05) (Table 3).

**Table 2:** Comparison of Birth Numbers in Mice Fed with Boric Acid Supplemented Diets

Group	Number of Dames	Number of offspring born	Offspring Per Dame	p
**Control**	6	55	9.17 ^aa^ ± 1.83	ns
**250 ppm BA**	6	51	8.50 ^aa^ ± 2.42	ns
**500 ppm BA**	6	52	8.67 ^aa^ ± 1.51	ns

^ns^ no significant difference (*p* > 0.05)

BA: boric acid

**Table 3:** Body Weight Changes (gr)

Group	Control	250 ppm BA	500 ppm BA	p
**Female Weight**	9.5 (9.25-11)	11.5 (9.0-12.25)	7.5 (7.5-9.0)	*p < 0.05 *p* = 0.0305, **250 BA & 500 BA**
**Male Weight**	10 (9.5-10.25)	10 (8.5-11.25)	8 (8-8.25)	*p < 0.05 *p* = 0.028, **C & 500 BA** *p < 0.05 *p* = 0.028, **250 BA & 500 BA**

*significantly different (*p* < 0.05)

BA: boric acid

For liver weight ratios, the measurements were recorded as follows: control female (4.54 ± 0.39), male (4.44 ± 0.25); 250-ppm BA female (5.05 ± 0.51), male (4.90 ± 0.32); and 500-ppm BA female (4.91 ± 0.47), male (4.17 ± 0.47). Statistical analysis revealed a significant difference in the liver and heart weight ratios of the 250-ppm BA female and male groups and the 500-ppm BA female group when compared to those of the control group (*p* < 0.05).

No significant difference in heart weight was observed between the 500-ppm BA male group and the control male group (*P* > 0.05) (Table 4).

**Table 4:** Calculation of Organ/Body Weight Ratios (%)

Sex	Organ	Control (C)	250 ppm BA	500 ppm BA	p
**Female**	**Liver (g)**	4.54 ± 0.39	5.05 ± 0.51	4.91 ± 0.47	ns
	**Kidney (g)**	0.68 ± 0.06	0.66 ± 0.22	0.85 ± 0.08	ns
	**Heart (g)**	0.62 ± 0.15	0.62 ± 0.13	0.87 ± 0.17	ns
	**Spleen (g)**	0.58 ± 0.06	0.73 ± 0.13	1.00 ± 0.24	**p < 0.001 *p* = 0.0051, **C & 500 BA**
**Male**	**Liver (g)**	4.48 ± 0.25	4.90 ± 0.32	4.17 ± 0.47	*p < 0.05 *p* = 0.0312, **250 BA & 500 BA**
	**Kidney (g)**	0.70 ± 0.19	0.62 ± 0.20	0.84 ± 0.12	ns
	**Heart (g)**	0.58 ± 0.11	0.58 ± 0.17	0.84 ± 0.10	*p < 0.05 *p* = 0.0233, **C & 500 BA** *p < 0.05 *p* = 0.0340, **250 BA & 500 BA**
	**Spleen (g)**	0.83 ± 0.09	0.83 ± 0.50	0.84 ± 0.16	ns

*significantly different (*p* < 0.05)

** significantly different (*p* < 0.001)

^ns^ no significant difference (*p* > 0.05)

BA: boric acid

The gender distributions of the born offspring were calculated in the control and 250- and 500-ppm BA groups. The numbers of born female and male offspring were 26, 35, and 35 and 29, 16, and 17, respectively. Thus, these gender distribution ratios were 47.44, 68.54, and 67.05% for females, and 52.56, 31.46, and 31.95% for males, respectively, with a statistically significant increase in female-biased offspring observed in the 250- and 500-ppm BA groups (*p* < 0.05) (Table 5).

**Table 5:** Ratios of Gender Distribution in Born Offspring

Group	Control (C) (*n* = 26)	250 ppm BA (*n* = 35)	500 ppm BA (*n* = 35)	p
**Female Percentage Ratio %**	47.44 ± 11.56	68.54 ± 15.08	68.05 ± 13.71	*p < 0.05 *p* = 0.0410, **C & 250 BA** *p < 0.05 *p* = 0.0462, **C & 500 BA**
**Male Percentage Ratio %**	52.56 ± 11.56	31.46 ± 15.08	31.95 ± 13.71	*p < 0.05 *p* = 0.0410, **C & 250 BA** *p < 0.05 *p* = 0.0462, **C & 500 BA**

*significantly different (*p* < 0.05)

BA: boric acid

Total antioxidant status (TAS) level measurements were conducted on the blood and organ samples obtained from the groups. The TAS levels of the serum samples of the control and 250- and 500-ppm BA groups were found to be (2.19 ± 0.18), (2.39*±0.18), and (2.39 ± 0.20), respectively. A significant difference in serum samples was detected in the 250-ppm group when compared to the control group (*p* < 0.05). In the heart samples, these measurements were 1.36 (1.25–1.70), 2.40 (1.96–2.61), and 2,29* (2,18–2,40)(); in kidney samples, they were (1.43 ± 0.2), (2.24 ± 0.23), and (1.95 ± 0.37)(); and in liver samples, they were 1.27 (1.18–1.34), 1.91 (1.60–2.12), 1.78 (1.56–1.98). Significant differences were thus observed in both the 250- and 500 ppm BA groups for all three organ samples taken from those of the control group (*p* < 0.05) (Table 6).

**Table 6:** Total Antioxidant Level of Serum and Organ Samples Obtained from the Groups

TAS Levels	Control (C)	250 ppm BA	500 ppm BA	P
**Serum**	2.19 ± 0.18	2.39 ± 0.20	2.24 ± 0.12	*p < 0.05 *p* = 0.0378, **C & 250 BA**
**Heart**	1.36 (1.25–1.70)	2.40 (1.96–2.61)	2.29 (2.18–2.40)	**p < 0.01 *p* = 0.0018, **C & 250 BA** *p < 0.05 *p* = 0.0144, **C & 500 BA**
**Kidney**	1.43 ± 0.21	2.24 ± 0.23	1.95 ± 0.37	***p < 0.001 *p* < 0.0001, **C & 250 BA** **p < 0.01 *p* = 0.0012, **C & 500 BA**
**Liver**	1.27 (1.18–1.34)	1.91 (1.60–2.12)	1.78 (1.56–1.98)	**p < 0.01 *p* = 0.0013, **C & 250 BA** **p < 0.01 *p* = 0.0037, **C & 500 BA**
**Spleen**	1.60 ± 0.37	1.75 ± 0.42	2.05 ± 0.46	Ns

*significantly different (*p* < 0.05)

** significantly different (*p* < 0.01)

*** significantly different (*p* < 0.001)

^ns^ no significant difference (*p* > 0.05)

BA: boric acid

When assessing the levels of the oxidative damage marker MDA in blood serum and organ samples obtained from the groups, the serum samples were measured as follows: control 2.28 (1.95–2.69), 250-ppm BA 0.94 (0.83–1.14), and 500-ppm BA 1.21 (1.02–1.46). The MDA levels in the serum, heart, kidney, and liver of the two BA groups were comparable but significantly lower than those of the control group (*p* < 0.001) (Table 7).

**Table 7:** Malondialdehyde (MDA) Levels in Serum and Organ Samples Obtained from the Groups

MDA LEVELS	Control (C)	250 BA	500 BA	P
**Serum**	2.28 (1.95–2.69)	0.94 (0.83–1.14)	1.21 (1.02–1.46)	***p < 0.001 *p* < 0.0001, **C & 250 BA** **p < 0.01 *p* = 0.0082, **C & 500 BA**
**Heart**	1.36 (1.25–1.70)	2.29 (2.18–2.40)	2.4 (1.96–2.61)	*p < 0.05 *p* = 0.0144, **C & 250 BA** **p < 0.01 *p* = 0.0018, **C & 500 BA**
**Kidney**	4.79 ± 0.72	2.24 ± 0.23	1.952 ± 0.37	***p < 0.001 *p* < 0.0001, **C & 250 BA** ***p < 0.001 *p* < 0.0001, **C & 500 BA**
**Liver**	5.64 ± 0.74	2.71 ± 0.70	2.33 ± 0.75	***p < 0.001 *p* < 0.0001, **C & 250 BA** ***p < 0.001 *p* < 0.0001, **C & 500 BA**
**Spleen**	2.06 ± 0.52	2.38 ± 0.72	2.11 ± 0.63	ns

*significantly different (*p* < 0.05)

** significantly different (*p* < 0.01)

*** significantly different (*p* < 0.001)

^ns^ no significant difference (*p* > 0.05)

BA: boric acid

To demonstrate the increase in oxidative molecules, the total oxidant status (TOS) level was similarly measured in blood serum and organs. In the serum samples, the values were 8.4 (7.72–8.81) for the control group, 6.49 (5.90–7.30) for the 250-ppm BA group, and 7.33 (6.89–7.75) for the 500-ppm BA group. A significant difference in serum sample from the control group was thus observed in the 250 ppm BA both BA group (*p* < 0.01). In organ measurements, the spleen values for the three groups were measured at 6.36 ± 0.65 (control), 6.44 ± 0.76 (250-ppm BA), and 7.87 ± 0.58 (500-ppm BA), showing a significant difference in the 500-ppm BA group (*p* < 0.001) (Table 8).

**Table 8:** Total Oxidant Status (TOS) of Serum and Organ Samples Obtained from the Groups

TOS Levels	Control (C)	250 ppm BA	500 ppm BA	P
**Serum**	8.4 (7.72–8.81)	6.49 (5.90–7.30)	7.33 (6.89–7.75)	**p < 0.01 *p* = 0.0024 **C & 250 BA**
**Heart**	7.09 ± 1.11	7.04 ± 0.92	7.19 ± 0.84	ns
**Kidney**	7.18 ± 1.11	6.40 ± 1.04	7.37 ± 0.80	ns
**Liver**	6.89 ± 0.79	6.77 ± 1.19	6.19 ± 0.68	ns
**Spleen**	6.36 ± 0.65	6.44 ± 0.76	7.87 ± 0.58	***p < 0.001 *p* < 0.0001 **C & 500 BA** ***p < 0.001 *p* = 0.0002 **250 BA & 500 BA**

*significantly different (*p* < 0.05)

** significantly different (*p* < 0.01)

*** significantly different (*p* < 0.001)

^ns^ no significant difference (*p* > 0.05)

BA: boric acid

To demonstrate, the total oxidant index (OSI) level was similarly measured in blood serum and organs. In the serum samples, the values were 3.84 (± 0.82) for the control group, 2.72 (± 0.55) for the 250-ppm BA group, and 3.27 (± 0.59) for the 500-ppm BA group. A significant difference in serum sample from the control group was thus observed in the 250 BA group (*p* < 0.05).

In organ measurements, the heart values for the three groups were measured at 5.21 ± 0.91 (control), 2.93 ± 0.93 (250-ppm BA), and 3.14 ± 0.57 (500-ppm BA), showing a significant difference in the heart samples from control group the 250 ppm (*p* < 0.01) and 500-ppm (*p* < 0.05) BA group (Table 8). The kidney values for the three groups were measured at 5.02 ± 1.1 (control), 2.86 ± 0.71 (250-ppm BA), and 3.78 ± 0.82 (500-ppm BA), showing a significant difference in kidney samples from the control group was the 250 ppm (*p* < 0.001) and 500-ppm (*p* < 0.01) BA group (Table 8). The liver values for the three groups were measured at 5.43 ± 0.60 (control), 3.54 ± 0.65 (250-ppm BA), and 3.48 ± 0.71 (500-ppm BA), showing a significant difference in liver samples from the control group was the 250 ppm (*p* < 0.01) and 500-ppm (*p* < 0.01) BA group (Table 9).

**Table 9:** Oxidative Stress Index (OSI) of Serum and Organ Samples Obtained from the Groups

OSI	Control (C)	250 ppm BA	500 ppm BA	P
**Serum**	3.84 ± 0.82	2.72 ± 0.55	3.27 ± 0.59	*p < 0.05 *p* = 0.0378. **C & 250 BA**
**Heart**	5.21 ± 0.91	2.93 ± 0.93	3.14 ± 0.57	**p < 0.01. **C & 250 BA** *p < 0.05 **C & 500 BA**
**Kidney**	5.02 ± 1.1	2.86 ± 0.71	3.78 ± 0.82	***p < 0.001 *p* < 0.0001. **C & 250 BA** **p < 0.01 **C & 500 BA**
**Liver**	5.43 ± 0.60	3.54 ± 0.65	3.48 ± 0.71	**p < 0.01 **C & 250 BA** **p < 0.01. **C & 500 BA**
**Spleen**	3.98 ± 0.68	3.68 ± 0.48	3.84 ± 0.52	ns

*significantly different (*p* < 0.05)

** significantly different (*p* < 0.01)

*** significantly different (*p* < 0.001)

^ns^ no significant difference (*p* > 0.05)

BA: boric acid

## Discussion

Understanding the compounds and capacities of antioxidants in foods, preserving them during processing, and enhancing them are essential components of a healthy dietary approach. Many studies have revealed the effects on the health of antioxidant compounds found in various foods. These compounds can protect our health by preventing damage caused by oxidative stress and slowing down the aging of cells. Some compounds, as observed in the case of BA, exhibit antioxidant effects, particularly through endogenous antioxidant enzymes. Such studies are crucial for forming a healthy dietary approach, and the effects on the health, performance, and metabolism of experimental animals should also be evaluated [16].

In this study, the pregnancy performance and developmental and biochemical effects of mice fed with a BA-supplemented diet (250 and 500 ppm) were investigated, and the values obtained were compared with a control group of mice fed with standard pellet feed. There was no statistically significant difference in the number of newborn individuals among the BA and control groups.

In another study, different boron content feeds were given to 40-week-old egg-laying hens for 120 days. No significant differences were found among the groups in terms of live weight, feed consumption, feed conversion ratio, egg yield, egg weight, or specific weight [17]. In other studies, it was determined that a dose of 1000 ppm BA exhibited toxic effects, leading to a decrease in fetal weight in mice, rats, and rabbits, and the dosage with non-observable side effects was determined at 750 ppm [18]. Here, there was no significant difference in the weights measured on the 21st day of newborn individuals between the BA and control groups. However, it was observed that 500 ppm of BA reduced feed consumption and weight gain.

In a study involving 30 rats, 100-mg/kg doses of BA or borax were added to rat feed, and the effects on lipid peroxidation, some vitamin levels, antioxidant activity, and DNA damage were investigated. After 28 days, it was determined that both BA- and borax-supplemented feeds corrected vitamin C levels, reduced lipid peroxidation levels, and significantly decreased MDA levels. Additionally, it was observed that they strengthened the antioxidant defense system [6].

The increase in oxidative molecules implies that the body produces more antioxidants to cope with oxidative stress. Therefore, the presence or absence of oxidative damage cannot be conclusively determined by measuring only TOS. Instead, evaluating the change in the TOS/TAS ratio allows for a more accurate determination of the presence of oxidative damage [19]. In our study, the TAS measurements showed that applications of 250 and 500 ppm BA gave heightened antioxidant activity in organs as compared to control groups. Also, we found in this research that the use of BA in feed significantly supported the antioxidant level of the offspring obtained by OSI index.

MDA levels are known to be used in detecting lipid peroxidation and to have a strong correlation with the degree of peroxidation [20]. In a study examining the effects of BA on erythrocyte MDA levels, it was found that BA added at low doses (5–50 mg/L) did not cause a significant change in MDA levels, but at a dose of 500 mg/L, a significant increase was observed [9]. Here, the MDA level, an oxidative stress marker, showed a significant decrease in the two BA groups as compared to the control group, while MDA levels of the two BA groups were similar.

Some epidemiological studies on individuals exposed to boron have been conducted. In a study examining the relationship between boron exposure and the Y:X chromosome ratio, an increased ratio of female births among workers with high boron exposure was found. This study identified a difference in the Y-chromosome ratio in the sperm of men exposed to high levels of boron, suggesting a decrease in the Y-chromosome and, consequently, a higher likelihood of female offspring. However, this study has some methodological issues, and the reliability of the results is uncertain. For example, it was conducted on a small sample group, thus limiting generalizability, while the impact of other factors (age, smoking, etc.) seems not to have been controlled [21]. In a study conducted in China, the numbers of male and female children of workers in control and boron exposure groups were compared [22]. The statistical analysis revealed no significant difference between the two groups in terms of the sex of their children. Overall, the effects of boron exposure on human reproductive health remain controversial and require further research. In our study, when comparing the gender distribution of the newborn mice, a statistical difference was found in the number of female offspring when BA groups were compared with control groups in terms of the same gender groups.

Gender selection is a highly complex issue. Maternal diet can influence the sex of offspring, as has been shown for female mice [23]. Another factor in this regard is that of birth order, which is still not fully understood. Some studies have suggested using ration improvements to enhance body condition as a method to influence the gender ratio of, for example, sheep [24]. The maternal feed additives BA can offer substantial benefits for farm animal breeding as breeding sexing diet. For example, breeding producers can focus on female offspring to ensure the growth of the flock and increase genetic diversity, while producers of animals for meat may prefer more male offspring, where the goal is to obtain male offspring that grow faster and have a higher meat yield with the target of obtaining offspring of the desired gender [25, 26].

Genetic methods used in some animal species involve modifying sperm or egg cells to carry a specific gender gene. Various factors, including ethical concerns, legal limitations, and scientific reliability, have to be considered regarding gender control, and further research and investigation are needed to both further enhance the productivity of the livestock sector and lead to the development of new methods in the future [27, 28]. In our research, the important point is that the BA feed group had a litter female-to-male gender ratio that was significantly higher than the control group. The use of such genetic methods in humans remains a highly controversial subject, of course.

Broadly, the data obtained from this experiment suggest that BA may have various positive effects on both animals and humans. The observed positive effects of BA on live weight gain and performance associated with feed consumption may be beneficial in the livestock industry. Regarding its effects on gender, research should be directed toward understanding the relationship between BA exposure and the Y:X chromosome ratio.

## Data Availability

No datasets were generated or analysed during the current study.
